# Stabilization of Colloidal Germanium Nanoparticles: From the Study to the Prospects of the Application in Thin-Film Technology

**DOI:** 10.3390/ijms242115948

**Published:** 2023-11-03

**Authors:** Viktoriia Slynchuk, Christine Schedel, Marcus Scheele, Andreas Schnepf

**Affiliations:** 1 Institute of Inorganic Chemistry, University of Tuebingen, Auf der Morgenstelle 18, D-72076 Tuebingen, Germany; viktoriia.slynchuk@uni-tuebingen.de; 2 Institute of Physical and Theoretical Chemistry, University of Tuebingen, Auf der Morgenstelle 18, D-72076 Tuebingen, Germanymarcus.scheele@uni-tuebingen.de (M.S.)

**Keywords:** germanium, nanoparticles, metastable, subhalide, thin film

## Abstract

We present the stabilization of halide-terminated Ge nanoparticles prepared via a disproportionation reaction of metastable Ge(I)X solutions with well-defined size distribution. Further tailoring of the stability of the Ge nanoparticles was achieved using variations in the substituent. Ge nanoparticles obtained in this way are readily dispersed in organic solvents, long-term colloidally stable, and are perfect prerequisites for thin-film preparation. This gives these nanomaterials a future in surface-dependent optical applications, as shown for the halide-terminated nanoparticles.

## 1. Introduction

**Figure 1:**
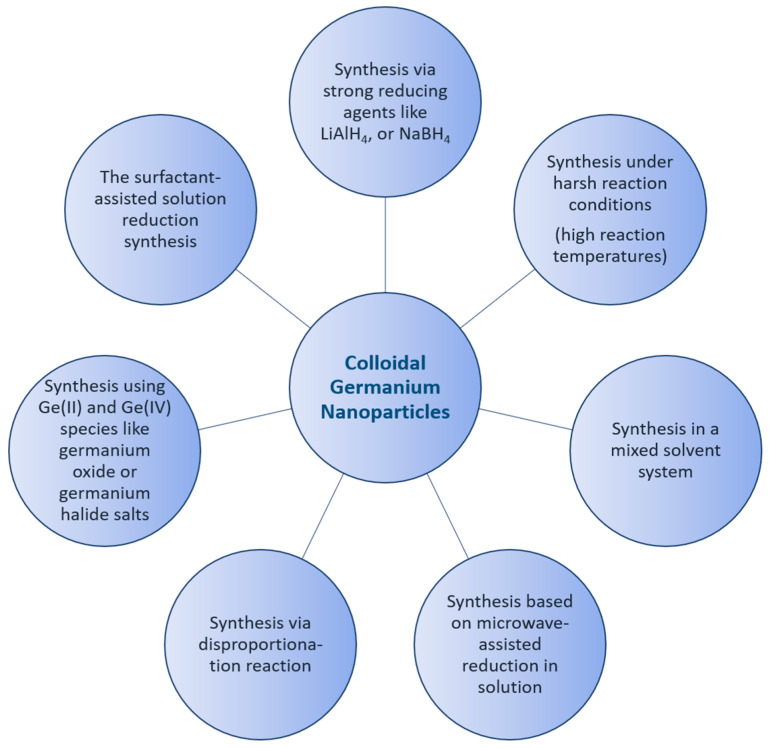
Schematic representation of the synthetic methods of colloidal Ge nanoparticles.

Germanium nanoparticles have been known and applied for a few decades in the form of colloidal solutions, and they have attracted great interest within the scientific community due to their physical properties and potential applications [1]. Along with this growing interest, sophisticated synthetic routes were developed to obtain germanium nanoparticles with different size distributions [2]. However, there are still many open questions. Currently, the main key issues in the synthetic roads to nanoparticles in general are the reproducibility and dispersion of nanoparticles [3]. This problem is, however, partially essential, as the synthesis is often prone to yielding particles with a broad distribution of sizes, shapes, and defects. The syntheses of inorganic nanoparticles in general have received growing attention in recent years, increasing reaction rates and yields, as well as leading to better protocols to give better selectivity and reproducibility. The state of the synthesis and the role of the surface modification of semiconductor nanoparticles, as well as the theoretical description of the size effect, have been reviewed [4,5]. Nanoparticle characterization is thereby a crucial step required to fully comprehend the origin of nanoparticle behavior and subsequently translate their performance benefits from laboratories into specific real-world applications [6,7]. The best preparation techniques for semiconductor nanoparticles come from colloid chemistry. One major reason for this is that there have been relatively mature methods for synthesizing size-controllable nanoparticles. In many cases, the size of semiconductor nanoparticles can be tuned by adjusting the concentrations of the surfactants, reaction temperature, and the duration of the particle growth. Additionally, a variety of chemical methods have been introduced for the synthesis of colloidal germanium nanoparticles, whereby the control of the properties of the synthesized nanomaterial is a key issue to their potential applications in different fields [8,9,10,11,12]. Most studies are focused on the synthetic routes following the pioneer works of Efros [13], Brus [14], and Henglein [15]. Currently, the key issue hindering the utility of nanoparticles in the industry is reproducibility. This problem might result from the dispersity and shape of the as-produced nanoparticles. The synthesis of II-VI semiconductor nanoparticles is already established, with great control of the size and surface termination of these nanoparticles [16,17]. Due to their complexity, the synthesis of III-V semiconductor nanoparticles is not as well established. Determining the properties of nanoparticles and exploring their structure is a critical challenge [18,19]. For example, colloidal germanium telluride nanoparticles have been used to study the dependence of phase stability on size. Thereby, both structural phase stability and phase transitions change dramatically in the nanometer size regime, where the surface plays a significant role in determining the overall energetics of the system [20]. Determining the physicochemical properties of nanoparticles and exploring their structure-property relationships are thus critical challenges [21]. In a colloidal solution, the impact of the optical properties of germanium nanomaterials is complex, strongly dependent on the preparation method, and explained by a combination of size and surface properties [22,23,24]. As a result, the optical properties of germanium nanoparticles have attracted interest in a range of photovoltaic applications [25,26]. A number of studies have investigated surface modification, and it played an important role in the stabilization of Ge nanoparticles. Additionally, the overall quality of the functional groups of nanoparticles played a dominant contributing factor for an application approach [27,28]. Over the last few years, the solution synthesis, surface passivation, optical properties, biomedical applications, and cytotoxicity of germanium nanoparticles have been compared and contrasted, and synthetic protocols have improved considerably. The wide properties and control of the surface chemistry of germanium nanoparticles make them highly suitable for a wide range of applications in different directions, including bio-imaging, light-emitting diodes, and solar cells [25,29,30]. Hence, a variety of methods have been developed to fabricate germanium nanoparticles, as summarized in the following Figure 1.

Thereby, applications of halide-terminated germanium nanomaterials are currently limited (Ge − X; X = Cl, Br, I) as the halide termination is air- and moisture-sensitive [31,32]. However, several multistep functionalization reactions have been successfully performed on germanium nanomaterials, producing a range of functional groups that are difficult to achieve with single-step reactions. For example, treating chloro-terminated germanium nanocrystals with a Grignard reagent yielded acetal-terminated germanium nanocrystals. Afterward, from acetal-terminated germanium nanocrystals, a whole range of unique functional groups was unlocked (hydroxyl, ester, acid) via treatment with appropriate reagents [33]. Germanium nanoparticles functionalized with Grignard reagents show similar photostability to alkyl-terminated nanoparticles formed via hydrosilylation/germylation. The reactions between halide-terminated nanoparticles and amines can strongly modify the optical properties of the nanoparticles. There is, thus, the possibility to passivate germanium nanoparticles with conjugated amines [34,35]. It is usually believed that proper synthetic methods, structure/property relations, and processing can direct the material to an application in a certain field. The study of amorphous and crystalline thin films obtained using different methods and modes is of great interest in the field of nanoelectronics. Thereby, nanoscale effects play a huge role in thin film fabrication, and there are a number of investigations that aim to obtain thin films and create photoelectric converters on their basis, which include clusters, nanowires, nanotubes, nanoparticles, fullerenes, endofullerenes, quantum dots, quantum wells, graphene, etc. [4,36,37].

**Figure 2:**
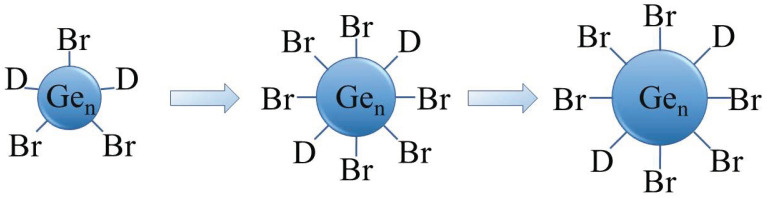
Schematic representation of the Ge nanoparticles size development (D = donor (P^n^Bu_3_)).

Recently, we presented a mild synthesis and characterization of halide-terminated colloidal Ge nanoparticles with a narrow size distribution, synthesized via a disproportionation reaction of metastable Ge(I)X solutions. The synthetic method is depicted as a simplified scheme in Figure 2 [38,39]. The size of the particles can be easily controlled using defined annealing times at certain temperatures. The Ge-Br-moieties at the surface of the particles open the door for further surface functionalization.

In this paper, we report the stabilization of as-synthesized Ge nanoparticles with further colloidal stability and precise size distribution from 4 to 25 nm. These nanoparticles are readily dispersed in organic solvents such as tetrahydrofuran (thf) and toluene, partially dispersed in ethanol and acetonitrile, and insoluble in hexane and pentane. The tuning of the germanium nanoparticles was achieved using variations in the substitution reactions. Moreover, the well-dispersed nanoparticles can be used for thin film preparation. Additionally, the absorption and the photoresponse of as-prepared thin films are studied.

## 2. Results and Discussion

**Figure 3:**
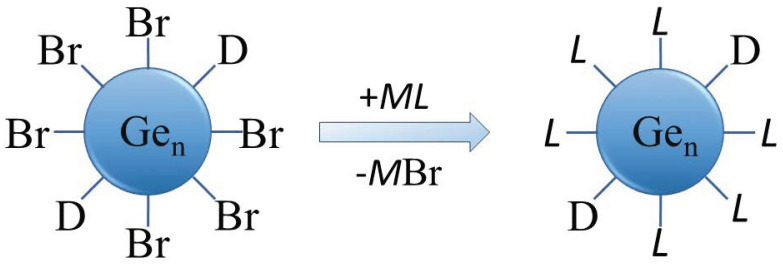
Schematic representation of the substitution of Br with organic or other functional groups for surface functionalization of the Ge nanoparticles (D = donor (P^n^Bu_3_), M = alkaline or alkaline earth metal, L = ligand).

The solubility and surface properties of the as-prepared halide-terminated germanium nanoparticles are key aspects in their synthesis (STEM image and size distribution in THF are presented in Figure S1, Supplementary Materials). The halide termination of the nanoparticles is a perfect prerequisite for further surface functionalization but also leads to the germanium nanoparticles having a high sensitivity to water and air. Additionally, the halide-terminated particles are thermally less stable, as evidenced by their degradation in transmission electron microscopy (TEM) studies. The as-synthesized, halogen-terminated nanoparticles can be readily functionalized due to the facile substitution of the halides via organic or other functional groups, which is one goal of the presented actual studies. The substitution might lead to a higher stability of the nanoparticles, being an important prerequisite for further applications and characterization. The substitution reaction thereby follows the reaction sequence outlined in Figure 3.

**Figure 4:**
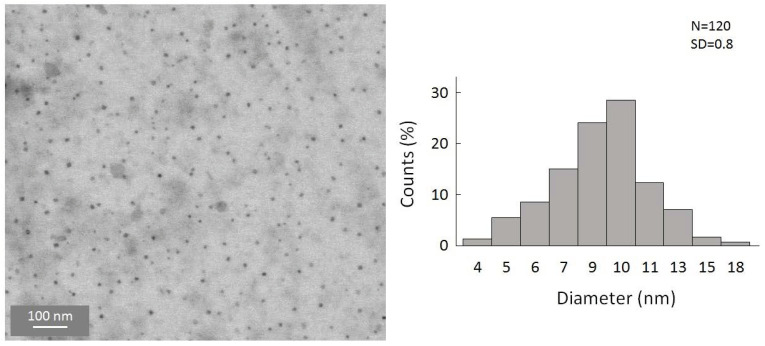
STEM images of amide-terminated germanium nanoparticles and size distribution of statistical evaluation of 120 nanoparticles on STEM images (with a size deviation of ±1 nm).

To gain a first insight into the reaction, the simplest organolithium reagents, such as CH_3_Li, (CH_3_)_3_CLi, and C_4_H_9_Li, were used primarily. However, these substitution reactions with the organolithium compounds led to a precipitation of the particles in a short time after the reaction. We were also not able to disperse these particles to, again, obtain a colloidal solution. Hence, a substitution with simple alkyl substituents is not useful to obtain colloidally stable, alkyl-terminated germanium nanoparticles. However, during these experiments, and the search for a suitable ligand that is able to keep the particles colloidally stable in solution, significant success was achieved when applying -N(SiMe_3_)_2_ as the substituent. Hence, the substitution reaction of the halide-terminated nanoparticles with the ligand source K[N(SiMe_3_)_2_] gives a nearly black-colored reaction solution and no precipitation of the substituted particles. Additionally, the substituted particles show a greatly increased stability, this time allowing, besides dynamic light scattering (DLS) measurements (Figure S2, Supplementary Materials), a characterization via TEM [40]. This is very important as scanning transmission electron microscopy (STEM) is one of the most important characterization techniques for nanomaterials [41,42]. Due to the thermal instability, these analytic techniques could not be used in the case of the halide-terminated nanoparticles. Consequently, a significant advantage of the synthetic route presented here is the high solution stability of the nanoparticles and their relatively narrow size distribution, which provides further opportunities for applications. The STEM images are presented in Figure 4, showing that uniform, non-agglomerated nanoparticles with a narrow size distribution are present. Most particles show a diameter of around 9–10 nm. However, some larger particles with diameters of up to 24 nm are also present, as well as smaller particles with a diameter of only 4 nm. The solution is thereby colloidally stable. To examine the influence of the solvent on the stability of the solution, THF was replaced with Toluene, leading to not only a significant difference in the short-term colloidal stability but also massively changing the long-term stability. Hence, the colloidal solution in THF is stable for over a year and in toluene only for a few months.

Besides the change in the properties, further evidence that the substitution reaction had taken place was the precipitation of crystals of KBr as the expected side product of the substitution reaction. The overall composition of the germanium nanoparticles was determined using energy-dispersive X-ray spectroscopy (EDX) and elemental analysis, as proof of the ligand exchange. (Tables S1–S4, Supplementary Materials). In the case of the EDX measurements, the result of the carbon content showed a high deviation as a carbon substrate was used. However, from the elemental analysis, it is obvious that, besides bromide substituents, some part of the PnBu_3_ donor is left on the surface of the nanoparticles and continues to act as a surfactant, as schematically shown in Figure 3. Additionally, the halide content of the particles was analyzed via titration with AgNO_3_, further showing that the halide atoms are not substituted quantitatively during the metathesis reaction. These remaining halide atoms might be substituted with another substituent, showing that further functionalization is possible here.

**Figure 5:**
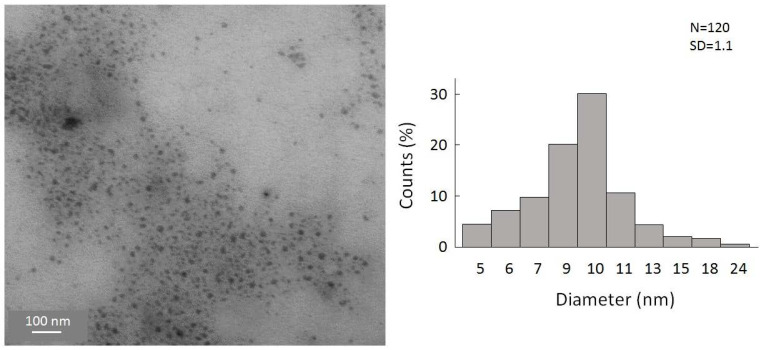
STEM images of cyclopentadienyliron dicarbonyl-terminated germanium nanoparticles and size distribution of statistical evaluation of 120 nanoparticles on STEM images.

In the case of the substitution reaction with the ligand source KFeCp(CO)_2_, it gives a dark red-colored reaction solution and increases the stability of the nanoparticles as well [43]. Consequently, analysis via STEM was possible this time, whereby the STEM images of cyclopentadienyliron dicarbonyl-terminated germanium nanoparticles showed, again, nanoparticles with a small agglomeration and a narrow size distribution (Figure 5). Additionally, EDX measurement of the particles (Table S2, Supplementary Materials) showed a lower halide content, hence, now, a nearly quantitative substitution of the halide atoms had taken place.

As both substitution reactions give small germanium nanoparticles (amide- and cyclopentadienyliron dicarbonyl-terminated) with comparable size and size distribution, this indicates that, also, the halide-terminated germanium nanoparticles should be of comparable size which was before shown with dynamic light scattering only.

**Figure 6:**
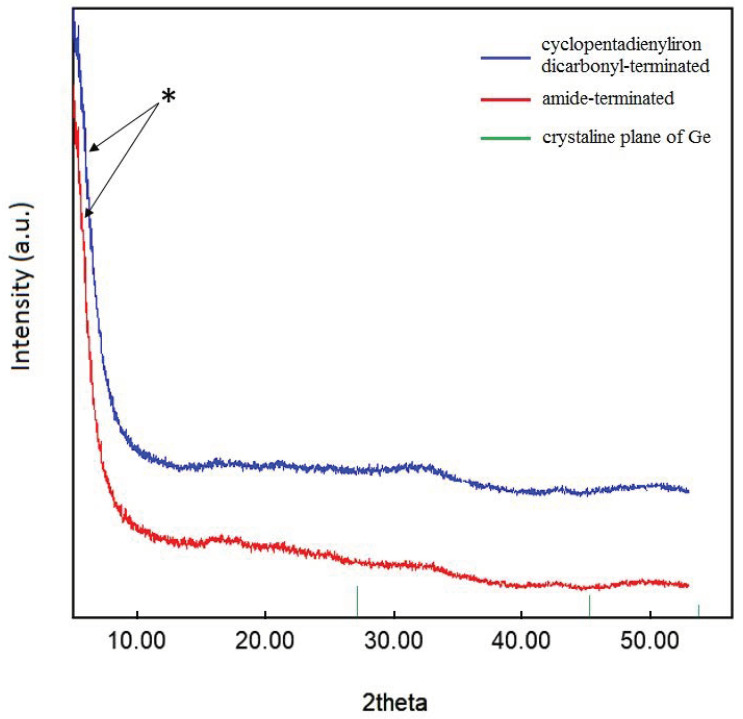
Powder XRD pattern of germanium nanoparticles. The star marks particle-particle correlations (see text). Crystalline plane of Ge reference: PDF # 00-004-0545.

Additionally, powder X-ray diffraction (PXRD) was used to characterize the crystallinity of the substituted nanoparticles, indicating that no crystalline core of, for example, α-germanium was present (Figure 6) [44,45]. Nevertheless, the width of the strongest peak (marked with a star in Figure 6) in the powder patterns indicated particle–particle coherency, in accordance with the previously reported [(P^n^Bu_3_)_0.3_Ge_1.8_Br]_n_ nanoparticles. The difference to the previously reported pattern indicates that this time, we have a new system with the possibility to measure intact samples. That is the reason for the absence of the peaks in the 2-theta range between 15 and 20°, which was observed before in terms of the sensitivity of the nanoparticles. In previous measurements on the halide-terminated germanium nanoparticles, the surface layer was partially oxidized to form mixed germanium-halide-oxide derivatives, resulting in increased intensity of impurity peaks. This then leads, after a prolonged measurement time, to small diffraction peaks that correspond to the GeO_2_ diffraction pattern, underlining the sensitivity of the halide-terminated particles to moisture and air as the GeO_2_ results from a reaction of the particles with a small amount of water or oxygen during the measurement [39]. The substitution of the halide atoms of the halide-terminated germanium nanoparticles thus not only leads to a strong increase in the thermal stability of the particles but also to a higher stability against moisture and air. Taking into account that the halide-terminated nanoparticles are, like the metastable Ge(I)Br solutions [46], sensitive to oxidation, further applications need to be performed under the exclusion of water and air. However, after the substitution, the particles are more stable, and, thus, further applications might be performed more easily without rigorous exclusions of moisture and air. Nevertheless, the right substituent must be chosen to give colloidally stable solutions; otherwise, aggregation and precipitation take place after the substitution. As the halide-, amide-, and cyclopentadienyliron dicarbonyl-terminated germanium nanoparticles are all colloidally long-term stable in solution, we wondered if these particles could be used for the preparation of thin films.

**Figure 7:**
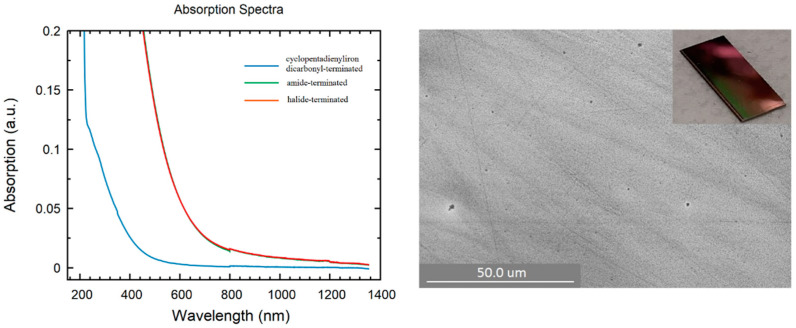
(**Left**): UV/Vis spectra of thin films: blue: cyclopentadienyliron dicarbonyl-terminated nanoparticles; green: amide-terminated nanoparticles; red: halide-terminated nanoparticles. (**Right**): SEM picture of substituted with halide-terminated germanium nanoparticles spin-coated thin film on a silica substrate. Inset: Snapshot of spin-coated film of halide-terminated germanium nanoparticles.

In addition to all previously mentioned applications of germanium nanoparticles, the interest in Ge thin film stems from the fact that Ge nanoparticles are used in thermal imaging, photodetectors, photovoltaic systems, and various other applications, such as solar cells, etc. [47,48,49,50,51]. Moreover, the use of relatively simple experimental equipment to create a Ge thin film allows one to obtain, study, and tailor their properties at a surprisingly low cost. However, this is challenging due to multiple issues such as strained films or high surface roughness, and there has been a lot of effort to grow high-quality thin films incorporating Germanium on Si substrates [52,53,54,55,56]. In this respect, different thin film samples of halide-terminated, amide-terminated, and cyclopentadienyliron dicarbonyl-terminated germanium nanoparticles were prepared by spin coating in an Ar-filled glovebox. The ability to form a thin film definitely opens the door to a new area of applications of these germanium nanoparticles and a promising route to improve the understanding of the properties of such synthesized nanoparticles. The SEM pictures directly show strong differences in the obtained films. In the case of the halide-terminated nanoparticles (Figure 7), a closed smooth film was obtained. In the case of the amide- and the cyclopentadienyliron dicarbonyl-terminated germanium nanoparticles, less perfect films with holes were obtained (Figure S5, Supplementary Materials). Considering the fact that there was a different color of the thin films, we checked the optical properties of the as-prepared films, which showed like the particles (Figure S3, Supplementary Materials) differences in the absorption spectra, depending on the surface termination.

Thereby, to obtain the UV/Vis absorption spectra, the films were prepared on a glass substrate and, afterward, the absorption of this film was measured and occurred in the case of the film of the cyclopentadienyliron dicarbonyl-terminated nanoparticles at wavelengths 300 and 450 nm (Figure 7). This absorbance was quite different with respect to the films of the halide-terminated and amide-terminated germanium nanoparticles, which were quite similar and showed absorbance at lower wavelengths between 500 and 850 nm. Hence, the substitution of the halide substituents partially changed the optical properties, which might be useful for future investigations. However, regardless of these features, the opto-electronic properties of the measured films were different, as shown in the following using electrical measurements.

**Figure 8:**
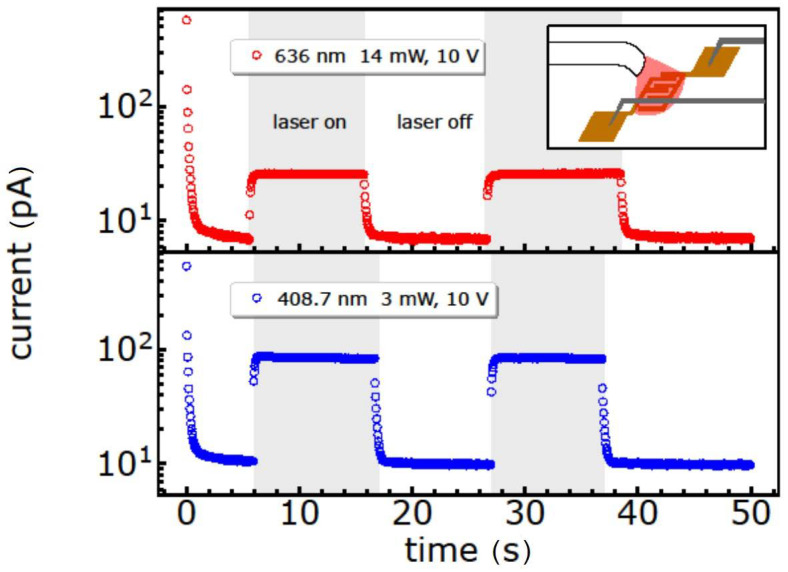
On/off photodetector properties of the GeBr nanoparticles under 636 nm (14 mW) and 408.7 nm (3 mW) laser illumination with a constant source-drain voltage of 10 V. Grey boxes indicate the ON-periods of laser illumination. Example of a 2.5 µm × 10 mm device. Inset: Schematic of the detector measurement.

As a result, the current response of the GeBr thin film towards 636 nm and 408.7 nm continuous wave laser illumination, under a constant source-drain voltage of 10 V, is shown in Figure 8 (Figure S4, Supplementary Materials). This illumination induces a photoresponse for both wavelengths with a higher response under blue laser illumination, as expected from the UV/Vis spectrum. The very small dark current, and the existing sub-bandgap response with an ON/OFF ratio of up to 10, suggest that these nanoparticles might be possible candidates for UV photodetectors when illuminated with laser energies. However, in the case of the other thin films prepared with nanoparticles functionalized via -FeCp(CO)_2_ and -N(SiMe_3_)_2_, all the samples were insulating and did not show a photoresponse. Such a result can occur, but not necessarily, in response to a sticky and insulating surface, e.g., due to the bulkier substituents N(SiMe_3_)_2_ and FeCp(CO)_2_ with respect to simple halide-termination. Furthermore, the strong reduction in the reflectivity of thin films in the visible-UV region is useful in photovoltaics, with special emphasis on multijunction solar cells [57].

## 3. Materials and Methods

All reactions were carried out under rigorous exclusion of air and moisture using Schlenk techniques under nitrogen or argon atmosphere. All organic solvents were dried over sodium and purified via distillation. DLS measurements were performed on a Malvern Zetasizer Nano ZS; Malvern Panalytical GmbH, Kassel, Germany. EDX analysis was performed at a HITACHI SU8030 scanning electron microscope; Hitachi Germany, Mannheim with Bruker-EDX; Bruker Corporation, Billerica, MA, USA, using solid samples, which were prepared in an Ar-filled glovebox. Elemental Analysis was performed using solid samples at a Vario Micro cube from Elementar Analysensysteme GmbH, Langenselbold, Germany. Powder XRD patterns were measured at room temperature with a Stoe STADI-P X-ray diffractometer; STOE & Cie GmbH, Darmstadt, Germany using monochromatized Cu-Kα1 radiation (λ = 1.540598 Å) and a Mythen–1K detector; STOE & Cie GmbH, Darmstadt, Germany. Standard measurements were taken in a q range of (0.2–6 Å^−1^). Powder samples were fixed under an argon atmosphere with grease between two Mylar foils.

### 3.1. Synthesis of the Particles

The nanoparticles were prepared via a disproportionation reaction of metastable subvalent Ge(I)Br solutions. Synthesis of the metastable Ge(I)Br solutions was performed as follows: liquid germanium (40 mmol) reacted with HBr (40 mmol) at approximately 1600 °C under high vacuum conditions (10^−2^ mbar). The resulting gaseous products are afterward condensed with 200 mL of a 10 to 1 mixture of THF/PnBu_3_ on a surface at −196 °C to give a solid matrix of the solvent molecules with the Ge(I)Br molecules embedded. Afterward, the solid matrix was warmed to −78 °C to give an orange-reddish solution. This metastable solution can be stored at −78 °C without decomposition. Upon heating the solution to room temperature, a disproportionation reaction (e.g., 4GeBr → GeBr_4_ + 3Ge) already occurred at low temperature due to the instability of the oxidation state +I of germanium. Hence, the synthesis can be conducted under mild reaction conditions, which is a significant advance in comparison to the hitherto known synthetic methods where harsher reaction conditions and additional reducing agents are necessary.

For the particle synthesis, the solution was slowly warmed up to room temperature under stirring and was slowly heated for 16 h at 55 °C. The heating process increases the diameter of the germanium nanoparticles up to 10 nm. The size of the nanoparticles can be adjusted by varying the temperature and heating time for the altering of the metastable Ge(I)Br solution, resulting in deep red solutions of germanium nanoparticles with a narrow size distribution. The main temperature limitation of this synthetic procedure is the boiling point of the solvent. The hydrodynamic diameter of the particles was measured via dynamic light scattering (DLS). After the nanoparticles had formed, all volatiles were removed under vacuum to give an oily reddish substance, which was washed up to six cycles with pentane and then dried under vacuum. Afterward, the obtained dark red powder was dissolved in THF and filtered through a filter cannula to remove the agglomerations of non-dispersible particles. The dissolution of the powder in THF gives a dark red colloidal solution with the before-chosen hydrodynamic particle diameter from 2 to 10 nm. The overall composition of the GeNP was determined via energy-dispersive X-ray spectroscopy (EDX) and elemental analysis (Supplementary Materials Tables S5 and S6), leading to an overall formula of [(PnBu_3_)_0.3_Ge_1.8_Br]_n_. For elemental analysis, EDX, and powder XRD, all volatiles were removed in vacuum yielding a red powder, which is very sensitive to air and water. This powder was afterward used for the substitution reactions.

### 3.2. Substitution Reactions

To a solution of [(P^n^Bu_3_)_0.3_Ge_1.8_Br]_n_ (4.8 mmol, 990 mg) in thf (60 mL), a solution of KFeCp(CO)_2_ (4.8 mmol, 1036.8 mg) was added. The resulting reaction mixture was slowly stirred overnight to give a slurry reaction solution. The solution was left overnight in a fixed position without stirring so that all insoluble KBr could sediment. Afterwards, the solution was filtered off from the KBr residue. Then, the solvent was removed under vacuum to give a dark red residue. Washing with pentane and extraction with toluene gave a black toluene solution (1905 mg, 93.9%).

### 3.3. Film Preparation

For the preparation of the GeBr photodetectors, commercially available Si-field-effect transistor substrates with 770 nm thermal oxide (bottom-gate: n-doped silicon, n = 3 × 10^17^ cm^−3^), gold interdigitated electrodes of 10 mm width (thickness: 30 nm), and varying channel length (2.5, 5, 10, 20 µm) of the Fraunhofer Institute for Photonic Microsystems, Dresden, Germany, were used. Thin film preparation was performed in a N_2_-filled glovebox, via spin coating conditions 75 rps/min during 30 s spin coater, 150 µL.

### 3.4. Electrical and Photocurrent Measurements

The prepared photodetector devices were transferred in a Lake Shore Cryotronics probe station CRX-6.5K under N_2_ via a home-built transfer arm to avoid contact with air. The optoelectronic investigations were performed in this probe station under a pressure of 2 × 10^−6^ mbar with a Keithley 2634B System Source Meter at room temperature. The electrodes were contacted with 50 Ω impedance-matched tungsten two-point probes. The photocurrent was measured under continuous wave illumination of the devices using a 636 nm laser (Taiko PDL M1, LDH-IB-640-B, PicoQuant; Berlin, Germany) and a 408.7 nm laser (LDC210C + TED200C, LP405-SF10, Thorlabs; Newton, NJ, USA). The powers of 14 mW and 3 mW were chosen as the maximum for 636 nm and 408.7 nm laser illumination, respectively. The laser spot was not focused in order to illuminate the whole electrode area.

## 4. Conclusions

The present investigation provides important insights into the nature and chemistry of germanium nanoparticles prepared via the disproportionation reaction of metastable subvalent Ge(I)Br solutions, which gives germanium nanoparticles in high yield with a narrow size distribution. Despite all of this work, little is known about the specific advantages of this method in comparison to classical nanoparticle synthesis. Through the sensitivity to air and moisture of the halide-terminated particles themselves, it is clear that the stability of those particles depends on the kind of active surface groups. In addition, the present data are consistent with the requirements for possible future applications, e.g., as UV photodetectors, whereas as-prepared germanium nanoparticles are chemically stable under inert conditions and the reactivity can be well-controlled at room temperature and instantaneously in liquid phase. It should also be noted that the chemistry of the disproportionation reaction of metastable subvalent Ge(I)Br solutions is very rich. Having all the advantages of liquid-phase synthesis and workup, these nanoparticles might become very interesting as starting materials in thin-film technologies. Thereby, the possibility to substitute the halide atoms within other ligands gives the possibility to alter the properties which, in the case of amide-and cyclopentadienyliron dicarbonyl-terminated particles, leads to higher stability but poorer properties with respect to opto-electronic applications, and could open up a novel avenue for both fundamental research and practical application. Nevertheless, further substitutions should be possible for altering the properties for future applications. Thereby, the presence of halide substituents in the case of the amide-substituted germanium nanoparticles opens the door for particles with different substituents. Also, reductive coupling of these partially substituted particles might be possible to give larger aggregates, which is part of the ongoing research in our lab.

## Data Availability

The datasets generated during and/or analyzed during the current study are available from the corresponding author on reasonable request.

## Supplementary Materials

The following supporting information can be downloaded at: https://www.mdpi.com/article/10.3390/ijms242115948/s1.
