# Supplementary material for: Stabilization of Colloidal Germanium Nanoparticles: From the Study to the Prospects of the Application in Thin-Film Technology
Source: Int J Mol Sci. 2023 Nov 3;24(21):15948. doi: 10.3390/ijms242115948 (PMC10649905; doi:10.3390/ijms242115948)
Supplement: Supplementary file 1 [file ijms-24-15948-s001.zip › ijms-2632874-supplementary.pdf]

Electronic Supplementary Information

Stabilization of Colloidal Germanium Nanoparticles: From the Study to the Prospects of the Application in Thin-Film Technology

Viktoriia Slynchuk [1], Christine Schedel [2], Marcus Scheele [2] and Andreas Schnepf [1,*]

[1] Institute of Inorganic Chemistry, University of Tuebingen, Auf der Morgenstelle 18, D-72076 Tuebingen, Germany
[2] Institute of Physical and Theoretical Chemistry, University of Tuebingen, Auf der Morgenstelle 18, D-72076 Tuebingen, Germany

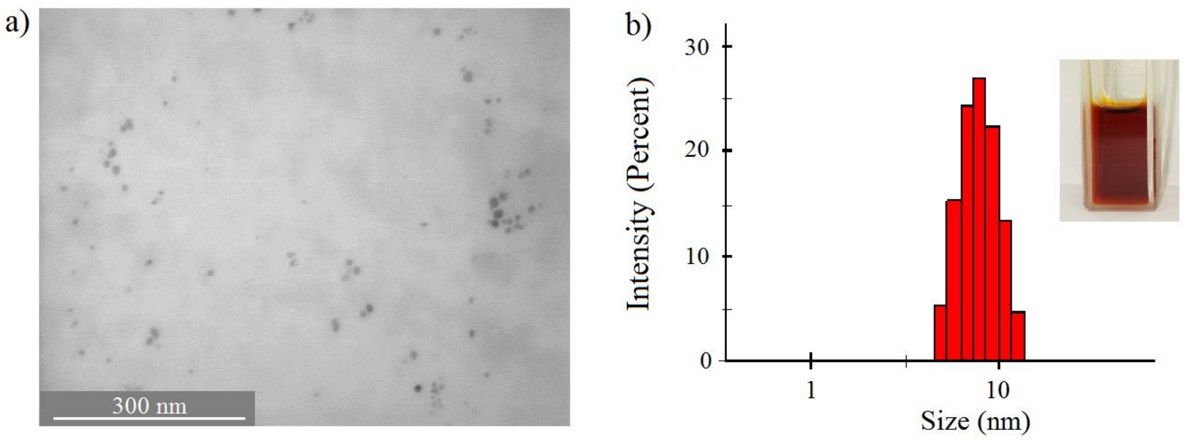

Figure S1. a) STEM image of halide-terminated germanium nanoparticles, b) Size distribution of halide-terminated germanium nanoparticles in THF measured by dynamic light scattering (DLS) at room temperature [1].

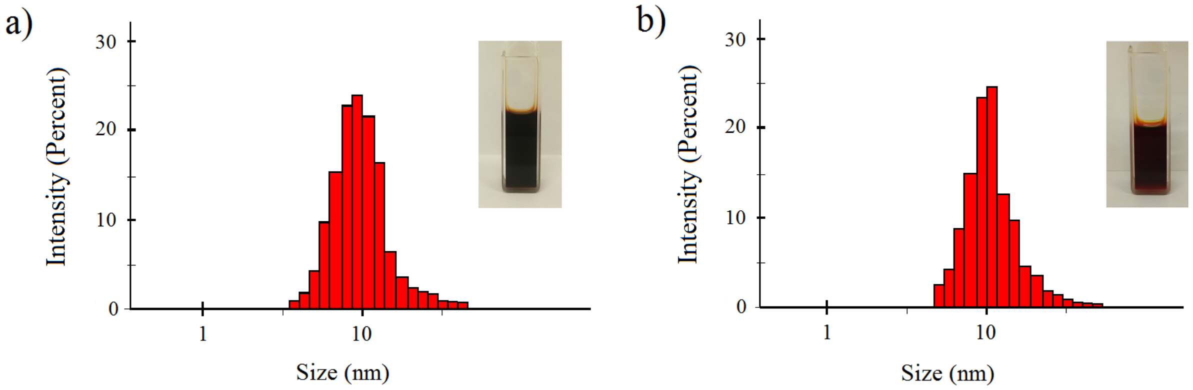

Figure S2. Size distribution of nanoparticles in THF measured by DLS at room temperature: (a) amide-terminated germanium nanoparticles, (b) cyclopentadienyliron dicarbonyl-terminated germanium nanoparticles.

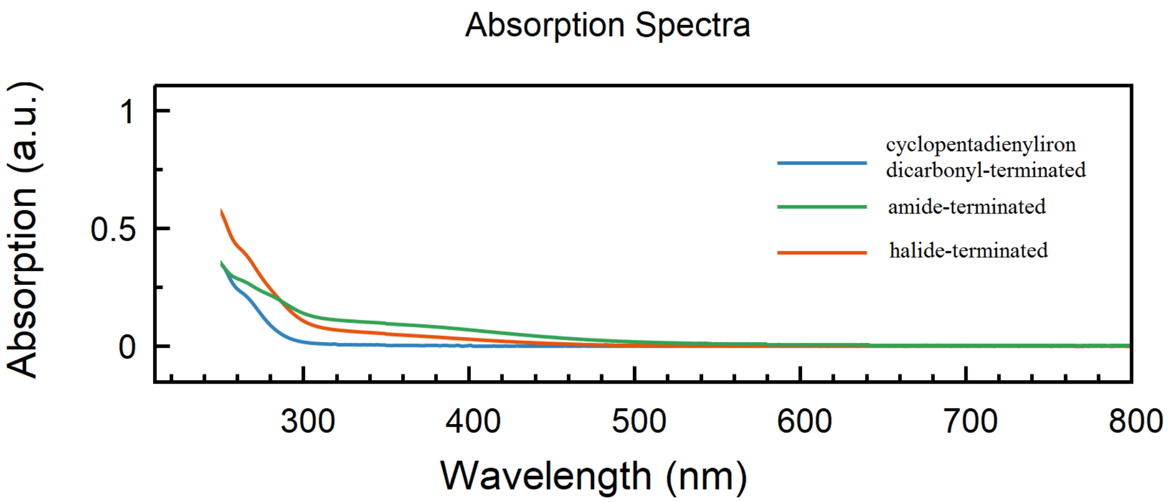

Figure S3. UV/Vis spectra of germanium nanoparticles exhibiting different substituents. Blue: cyclopentadienyliron dicarbonyl-terminated; Green: amide-terminated nanoparticles; Red: halide-terminated nanoparticles corresponding red UV/Vis spectrum.

The overall composition of the germanium nanoparticles was determined by energy-dispersive X-ray spectroscopy (EDX) and elemental analysis. The carbon substrate was used for the EDX measurements which is increasing the carbon evidence in the sample [2].

Table S1. Results of the EDX measurements of the amide-terminated germanium nanoparticles in a powder form on a carbon substrate

Element	[wt.%]	[norm.wt.%]	[norm. at.%]	Error in wt.% (3 Sigma)
Carbon	28.5	25.4	52.7	7.1
Germanium	40	43.2	19.2	2.9
Phosphorus	2.9	3.3	3.6	0.4
Oxygen	1.6	1.8	3.4	0.8
Nitrogen	3.4	4.1	5.4	0.2
Silicon	6.2	5.2	8.2	0.9
Bromine	13	16.2	6.4	1.8
Sum:	95.6	99.9	98.9	

Table S2. Results of the EDX measurements of the cyclopentadienyliron dicarbonyl-terminated germanium nanoparticles in a powder form on a carbon substrate

Element	[wt.%]	[norm.wt.%]	[norm. at.%]	Error in wt.% (3 Sigma)
Carbon	42.4	43.2	68.5	7.5
Germanium	24.7	23.0	6.0	4.0
Phosphorus	2.1	2.0	1.2	0.3
Oxygen	14.5	16.2	19.3	6.6
Iron	12.7	10.0	3.7	1.9
Bromine	2.8	4.5	1.0	0.7
Sum:	99.2	98.9	99.7	

Table S3. Results of the elemental analysis of the amide-terminated Ge nanoparticles

Element	[%]
Nitrogen	0.54
Carbon	20.81
Hydrogen	5.51
Sulfur	0

Table S4. Results of the elemental analysis of the cyclopentadienyliron dicarbonyl-terminated germanium nanoparticles

Element	[%]
Nitrogen	0
Carbon	36.95
Hydrogen	4.80
Sulfur	0

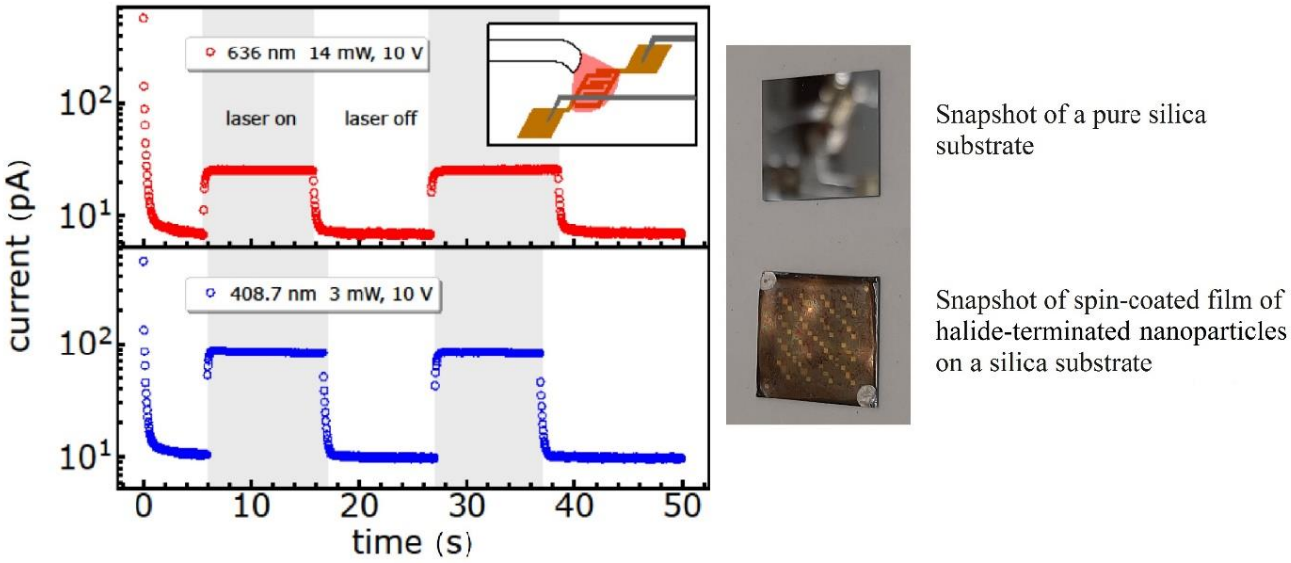

Figure S4. On/off photodetector properties of the GeBr nanoparticles under 636 nm (14 mW) and 408.7 nm (3 mW) laser illumination with a constant source-drain voltage of 10 V. Grey boxes indicate the corresponding laser illumination. Example of a 2.5 µm × 10 mm device. Inset: Schematic of the detector measurement.

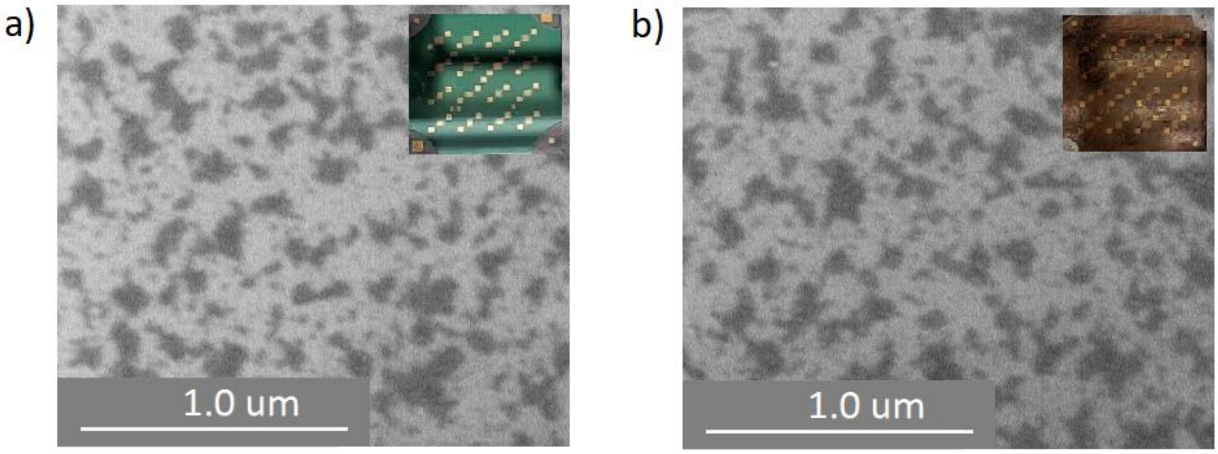

Figure S5. SEM picture of: a) amide-terminated germanium nanoparticles spin-coated thin film on a silica substrate (inset: snapshot of spin-coated film of amide-terminated germanium nanoparticles), b) cyclopentadienyliron dicarbonyl-terminated germanium nanoparticles spin-coated thin film on a silica substrate (inset: snapshot of spin-coated film of cyclopentadienyliron dicarbonyl-terminated germanium nanoparticles).

The overall composition of halide terminated germanium nanoparticles was determined by energy-dispersive X-ray spectroscopy (EDX) and elemental analysis [1]. The carbon substrate was used for the EDX measurements which is increasing the carbon evidence in the sample.

Table S5. Results of the EDX measurements of the halide terminated germanium nanoparticles powder

Element	[wt.%]	[norm.wt.%]	[norm. at.%]	Error in wt.% (3 Sigma)
Carbon	15.2	15.8	51.7	5.6
Germanium	46.0	47.7	26.0	7.4
Phosphorus	3.3	3.4	4.3	0.4
Oxygen	0.9	0.9	2.2	0.5
Bromine	31.0	32.2	15.8	4.2
Sum:	96.4	100	100	

Table S6. Results of the elemental analysis of the halide terminated germanium nanoparticles powder

Element	[%]
Nitrogen	0.002
Carbon	17.99
Hydrogen	3.44
Sulfur	0

References

1. Slynchuk, V.; Hodas, M.; Naglav-Hansen, D.; Schreiber, F.; Schnepf, A. New horizons for the synthesis of nanoparticles: Germanium nanoparticles from metastable GeBr-solutions. *Main Group Met. Chem*. **2021**, *44*, 243-249. https://doi.org/10.1515/mgmc-2021-0026
2. Modena, M. M.; Rühle, B.; Burg, T. P.; Wuttke, S. Nanoparticle Characterization: Nanoparticle Characterization: What to Measure? *Adv. Mater.* **2019**, *31*, 1901556. https://doi.org/10.1002/adma.201970226
